# Global health diplomacy in Mexico: insights from key actors in the field

**DOI:** 10.1186/s12992-021-00789-y

**Published:** 2021-12-02

**Authors:** German Guerra, Emanuel Orozco, Paulina Jiménez, Arne Ruckert, Ronald Labonté, Nelly Salgado de Snyder

**Affiliations:** 1 Centre for Health Systems Research, Global Health Program, National Institute of Public Health, Mexico City, Mexico; 2 School of Epidemiology and Public Health, University of Ottawa, Ottawa, Canada

**Keywords:** Global Health Diplomacy, Mexico, Stakeholders, International Relations, Foreign Policy

## Abstract

**Background:**

Global health diplomacy (GHD) focuses on the actions taken by diverse stakeholders from different nations –governments, multilateral agents, and civil society– to phenomena that can affect population health and its determinants beyond national borders. Although the literature on conceptual advancements of GHD exists, empirical studies about how health becomes an issue of relevance for foreign policy are scarce. We present an analysis of the entry processes of health into the foreign policy and diplomatic domains in Mexico from the perspective of key informants of three different sectors.

**Methods:**

A purposive sample of high-rank representatives of three sectors involved in GHD was designed: Two from Health Sector (HS), four from Foreign Affairs Sector (FAS), and three from Non-governmental organizations (NGOs). Nine semi-structured interviews were conducted exploring the topics of: (1) Health concerns entering diplomatic and foreign policy; (2) Processes that allow actors to influence foreign policy and negotiation and; (3) Impact of multilateral negotiations on decision-making at the national level.

**Results:**

Our analysis suggests that GHD in Mexico is hierarchically driven by the FAS and health concerns only enter foreign policy when they are relevant to national priorities (such as trade or security). HS possesses a lesser degree of influence in GHD, serving as an instance of consultation for the FAS when deciding on health-related issues at global meetings (i.e., World Health Assembly). NGOs resort to lobbying, advocacy, networking, and coalition-working practices with other sectors (academy, think-tanks) to prevent harmful impacts on local health from multilateral decisions and as a mean to compensate its power asymmetry for influencing GHD processes in relation to the government.

**Conclusions:**

GHD in Mexico occurs in a context of asymmetric power relationships where government actors have the strongest influence. However, NGOs’ experience in raising awareness of health risks needs to be weighted by government decision-makers. This situation calls for capacity building on intersectoral communication and coordination to create formal mechanisms of GHD practices, including the professionalization and training on GHD among government agencies.

**Supplementary Information:**

The online version contains supplementary material available at 10.1186/s12992-021-00789-y.

## Background

Global health diplomacy (GHD) describes how governments, multilateral agents, and civil society organizations from different nations respond to relevant situations to population health and its determinants, which transcend national borders. [1] As such, its practice straddles foreign policy and public health and is best understood by reference to disciplinary theories that inform both areas of knowledge. Core issues to GHD include global health risks, national health security, emergency health preparedness, and trans-border humanitarian health assistance. [2] Consequently, GHD can be understood as a space of confluence, negotiation, and debate between countries with different power levels to influence decisions to improve or sustain global health.

In the Americas, and specifically in Latin American countries, GHD is still a relatively new and heterogeneous field of study at the intersection of theory and practice, which calls for a better understanding of the specific contexts in which health arises as a foreign policy issue. [3] Some globally shared matters susceptible to become drivers of GHD for the region are securitization, trade, and implementation of global agendas for health, generally arising through intergovernmental processes or agreements. [4] For the Mexican case, emigration to the United States (US) and immigrant’s health have been the main drivers of GHD, as reflected by the existence of bi-national specialized bureaucracies[5] attending this matter, such as the US-Mexico Border Health Commission (CSF, acronym in Spanish),[6] and the Institute for Mexicans Abroad, a decentralized agency of the Mexican Secretariat of Foreign Affairs (SRE) to support Mexicans who live and work abroad,[7] which operates a longstanding consular program for healthcare access and promotion for Mexican immigrants in the US (*Ventanillas de Salud* or Health Windows). [8] Other GHD issues in Mexico besides immigration are trade affecting food policy and reproductive rights, [9, 10] but relatively little is known about how health issues enter foreign policy agendas in the first place. To address this knowledge gap, we examine how and why health matters are endorsed and integrated into foreign policy from the perspective of representatives of three major sectors in Mexico: Health, Foreign Affairs, and Non-Governmental Organizations. Finally, we identify some barriers to effective GHD in Mexico and provide recommendations for overcoming them when health diplomacy has garnered a renewed focus amidst the COVID-19 pandemic.

## Methods

This article reports the findings from a qualitative study included as a component of a larger multi-centric research project aimed at the empirical analysis of the GHD in four countries in the Americas region (Brazil, Canada, Chile, and Mexico), [11] integrating a range of theoretical frameworks from different disciplines. This comprises scholarly approaches to health as an international relations concern that include policy framing analysis, based on work by Labonté and Gagnon [4]; high-low politics dichotomy and classification of approaches for foreign policy (including realist and liberal outlooks) borrowed from international relations theory, based on work by Fidler [12, 13]; the application in foreign policy of the Kingdon’s empirically-based multiple streams model of policy adoption by Gagnon [14]; and the policy diffusion approach by Simmons and Elkins [15]. Among the main premises and core assumptions derived from these theoretical frameworks are: GHD as “niche diplomacy”; health entering the foreign policy sphere through securitization and as part of competition within the bureaucracy (i.e., bureaucratic politics); [16] a relationship of subordination of health (low politics) to security interests (high politics); multisectoral engagement and multilateralism as key factors for agenda setting and prioritizing within GHD processes; [17] and the conceptualization of actors as stakeholders *“who have an interest in the issue under consideration, who are affected by the issue, or who – because of their position – have or could have an active or passive influence on the decision-making process.“*[18].

The qualitative study was conducted between October 2017 and January 2018. We designed and chose a purposive sample of high-rank representatives of three sectors involved in GHD in Mexico: health, foreign affairs, and NGOs. Key informants (KIs) were identified based on previous literature reviews and document analysis, which informed our analytical framework, and other findings obtained in parallel phases of the research project. [11, 19–24] Contact with KIs was established using public government organizations’ directories, existing personal contacts, and purposive sampling using snowball technique. (See Table 1 for the final number and description of KIs).

Nine semi-structured interviews were conducted using an interview guide exploring the following topics: (1) Health concerns entering diplomatic and foreign policy; (2) Processes that allow actors to influence foreign policy and negotiation; and (3) Impact of multilateral negotiations on decision-making at the national level. The explored topics were based on a previously conducted review of literature and official documents related to GHD in Mexico, which aided in the triangulation of findings from empirical data.

Interviews were audio-recorded, transcribed, and coded into framework matrices based on a deductively developed code-tree by a team of researchers (see supplementary material), using QSR NVivo 10 software. Verbal informed consent was obtained from all KIs, ensuring the protection of their autonomy, anonymity, and confidentiality. The research project was approved by the Research Ethics Committee of the National Institute of Public Health, Mexico.

**Table 1:** KEY INFORMANTS INTERVIEWED

Informant code	Sector	Institution
A	Foreign Affairs	Former Mexican Ambassador
B	Foreign Affairs	Mesoamerican Project and member of the Mexican Agency for International Development Cooperation
C	Foreign Affairs	Permanent Mission of Mexico to the United Nations Office in Geneva
D	Foreign Affairs	Permanent Mission of Mexico at the United Nations in NYC
E	Health Sector	Foreign Affairs at the Secretariat of Health
F	Health Sector	US-Mexico Border Health Commission
G	NGO Sector	“Consortium for Parliamentary Dialog” [*Consorcio para el Diálogo Parlamentario*]
H	NGO Sector	The Power of the Consumer [*El Poder del Consumidor*]
I	NGO Sector	Coalition Counterbalance [*Coalición Contrapeso*]

In our analysis, we consider foreign policy as a bi-directional flow of processes, where decisions, consensus, or even disagreements taking place at the global level can influence domestic contexts (outside-in logic) and, vice-versa, national-level agendas, policies, and priorities can be positioned at the global level (inside-out logic), thus influencing the global governance landscape. [25]

## Results

This section describes KIs’ perceptions of the most relevant health-related concerns that enter foreign policy in Mexico, the processes that allow such entrance, and their impacts at the national level. We argue that these processes’ outcomes result from an underlying execution of strategies for power influence between sectors and stakeholders at the national level, aimed at prioritizing their own sectoral interests. This seems to be a scaled-down version of the exercise of power between nation-states commonly found in the global arena of multilateralism. The main findings are summarized in Table 2.

**Table 2:** GHD IN MEXICO: CONCERNS, PROCESSES, IMPACTS, AND SCOPE OF POWER BY SECTOR

SECTOR	CONCERNS		PROCESSES		IMPACTS	SCOPE OF POWER *
	Inside-Out	Outside-In	Strategies	Mechanisms		Power of priority setting in global agenda (+/++/+++)
**Health**	Border Health and Bi-National Health Health expenditure and financial protection (migrant health) Regional integration for migrant health	Global health Security Tobacco control	N/A	Specialized bureaucracies: · CSF · *Ventanillas de Salud* · Mesoamerican Health System · Global Health Security Initiative · Office for Tobacco Control in Mexico	Pressure from both private and public actors (pharmaceutical, food industry and other Secretariats of State) can take place if interests are affected.	+++ Northern Border Health (Bi-lateral) agenda ++ Southern Border (Mesoamerican Project) + Multilateral agenda
**Foreign Affairs**	National security against pandemics Vector-borne diseases Cooperation for development, regional integration, and health systems strengthening	Maternal and child health Road safety Primary health care Antimicrobial resistance Drug production, traffic, and consumption prevention	Mobilization of technical and political instruments developed by the UN agencies system Intersectoral consultation and negotiations (Academia, HS, industry) Creation of *ad hoc* intersectoral groups to define positions in foreign policy	Specialized bureaucracies: • Representations of Mexico at the UN System in Geneva (WHO) and NYC (Permanent Mission) • Mexican Agency for International Development Cooperation • Mesoamerican Project	When adhered agreements are not legally binding but can affect local sectors, their implementation is almost impossible due to intense pressure from affected parties.	+++ Decision about national policies against pandemics ++ Border health + Negotiation in international treaties + Sanitary restrictions in customs ++ Participation in international health forums + National health policy decision-making
**NGO**	Chronic diseases, tuberculosis, health financing, and universal health care	Women rights, obesity prevention, and road safety issues Frontal labeling of food packaging Special Tax on Production and Services for sugar-sweetened beverages	Strengthen positions by coalition-forming with other NGOs Political visibility and public agenda positioning Dissemination of activities in independent media outlets Serving as liaisons with political actors in health and foreign policy Expert and scientific evidence -based discourse and arguments	Partnerships with key actors, strengthening local institutional capacity Dialogue with government agencies at the local level Technical cooperation and funding through international organizations and donors Dissemination of health-related issues for broad audiences Identification of political windows of opportunity for agenda topic prioritization	Mexico signs and adheres to numerous agreements and global initiatives, however unlike trade treaties, the non-legally binding agreements are difficult to adapt and translate into local policies due to lack of enforcing mechanisms to comply and pressure from private actors	+ Decision-making processes +++ Effective in advocating health-related issues in foreign policy

+++Strong: Sector has the most decision-making power over agenda; intersectoral coordination is secondary to decision-making

++Moderate: Sector requires intersectoral coordination/consensus to exercise decision-making power

+Weak: Sector is mostly out of negotiations and requires formal invitations from other sectors to exercise decision-making power or participate in priority setting processes

* Note: “Scope of power” of sectors was inferred by considering the actor’s involvement with the topic, access to resources, and access to media. Additionally, we considered interview data and researchers’ group discussions until arriving at a consensus on each sector’s three levels

### Health concerns entering diplomatic and foreign policy

#### Health Sector (HS)

The most relevant GHD experience for the Mexican HS is found in its leadership to promote *bi-national health* within the CSF^1^, where health priority setting for border-living populations is jointly conducted by the health ministries of ten border states in Mexico (Baja California, Sonora, Chihuahua, Coahuila, Nuevo Leon, and Tamaulipas) and the US (California, Arizona, Texas, and New Mexico). Another important concern for the HS is healthcare access beyond Mexican national boundaries, aiding the Mexican population living in the US and other Latin American groups. This action has been mainly driven by the *Ventanillas de Salud* program operating at the 50 Mexican consulates in the US. These two examples have been widely recognized by KIs and in the relevant literature [8] as good practices for regional integration through migrant health promotion.

*“The contribution of Mexico in the resolutions on access to health services for the migrant and refugee population has been very important. It represents an example of good practices to make further proposals for attention to immigrants’ health in other countries that have no experience on the matter.“***(Informant F, Health Sector)**.

The HS experience regarding health concerns entering the national level has been channeled through the adoption and implementation of *global health initiatives*, such as the WHO Framework for Tobacco Control or *health securitization*, for instance, with its participation in the G7+Mexico Global Health Security Initiative in 2001, mainly due to its geostrategic location in the region of the Americas.

*“One of the main issues in which we are interested is global health security. It has allowed Mexico to be part of the G7+Mexico, which would be unthinkable if we did not have a border with the United States. So, we belong to this group called “Global Health Security Initiative” that comprises the seven most powerful countries in the world, plus Mexico.“***(Informant E, Health Sector)**.

#### Foreign Affairs Sector (FAS)

The FAS in Mexico does not maintain an office, department, or specific representation on health within its organizational structure. For this reason, conflicts and opposite positions on health issues may arise between the SRE and the Secretariat of Health (HS). There are no clear or direct linkages that are openly identifiable between both secretariats for collaboration on specific health issues. Furthermore, the FAS acknowledges that the Mexican HS have limited formal representation in the global UN agency system:

*“We do not have a representative from the Secretariat of Health in Geneva; we do all from the Secretariat of Foreign Affairs. Only when the level of complexity of some* [health] *issues is high, it does make it necessary to have more space for training* [capacities].*“***(Informant C, Foreign Affairs Sector)**.

One of the informants identified a lack of communication between SRE and HS as a barrier for better coordination. Nevertheless, some intersectoral collaborative opportunities have taken place when responding to sanitary emergencies, such as a dengue epidemic that broke out in Honduras. However, the communication from the Mexican embassy with the HS was not successful at providing the needed response. It was not until the problem was transferred to the SRE in Mexico to deal directly with HS that adequate interventions were carried out.

*“I tried to communicate with the Secretary of Health* [but] *he did not take the call. Then, I resorted to [*closer persons] *at the* [SRE], *and I told them about this very serious problem. Then they spoke to the Health Secretariat, and* (…) *they went to Honduras to start the whole fumigation and made a large donation of insecticide (Abate*, temephos larvicide*). It was through the SRE that the HS took action.“***(Informant A, Foreign Affairs Sector)**.

#### Non-Governmental Organizations Sector (NGO)

The NGO sector activities in the realm of GHD have primarily focused on policy issues surrounding food regulations in Mexico. NGOs have managed to lobby for, and position matters on food policy at global and national levels. However, incorporating issues such as pushing for the implementation of a *tax on sugar-sweetened beverages* or the *proposal of food frontal labeling* into national agendas were challenging, as NGOs confronted powerful commercial interests that limited or blocked their participation.

*“*[Since the beginning of the Trump administration] *there was an initiative by the US government in the renegotiation of NAFTA to include an article that would prevent any of the three countries from implementing a frontal warning type of labeling*^2^ (…) [When] *we learned about this, we denounced it, but also* [we learned that] *it was agreed with the Mexican government* [and that] *these negotiators were the same lobbyists of the industry* (…) *of food and beverages.“***(Informant H, NGO)**.

The NGO informants argue that health concerns are not considered relevant enough by government actors in global negotiations, especially when the implementation of global policies at the national level, related to prevention policies, conflicts with the economic interests of private actors. Furthermore, there have been situations in which another government agency is the one that promotes negotiations. For example, when the *Special Tax on Production and Services for sugar-sweetened beverages* was proposed in Mexico, it was surprisingly supported by the Secretariat of Finance and Public Credit (SHCP), who intervened for its implementation. This instance speaks of the competing interests of different government sectors.

### Processes allowing actors to influence foreign policy and negotiation

#### Health sector

The HS’s influence on GHD strongly depends on the area of policy engagement, either at the bilateral or multilateral level, with HS arguably taking a more passive role in the latter than the former. The HS’s involvement with foreign policy processes is largely determined by its relationship with the FAS. When it comes to the multilateral agenda, the FAS usually proposes the initiatives and the HS reacts to them. Consequently, in health-related multilateral agendas, the FAS assigns the HS a technical role for assisting Mexico’s resolutions and official positions in global meetings. This situation limits the work of the HS because generally, there are no representatives from this sector attending high-level international meetings, but the HS can occasionally have some influence, especially when targeting specific topics of national relevancy.

*“In the multilateral sphere, the one driving the questions of the integration of the foreign policy and the health components is the SRE so let us say that we are reactive* (…) [An example of an initiative by SRE was when] *we began to propose approaching trilateral health issues of Canada*, [US] *and Mexico. They* [the SRE] *responded to an initiative of* [US] *in terms of being able to maintain good security, health, epidemiological surveillance, strengthening cooperation, etc., and we* (…) *added the issue of childhood obesity in this trilateral agenda.“***(Informant E, Health Sector)**.

The agreed coordinated and collaborative work between the HS and the FAS around specific topics was identified as the most effective mechanism for including health topics in the foreign policy agenda. Typically, the HS prepares and provides the FAS with technical information, data, and relevant documents on health topics, allowing the FAS to define a national position in international declarations and multilateral forums.

*“The concrete mechanism is that we can develop a document and be able to negotiate it with SRE so that* [they] *can integrate it into a multilateral declaration. For example, the case of the Mesoamerican Declaration on Health and Migration* (…) *SRE gives us the information: the background framework, all the documents we have to refer to make this position more solid* (…) *It is a lot of work where we sometimes provide the substance, but they give us everything* (…) *the framework in which we must register this specific issue of health and migration in the case of our region.“***(Informant E, Health Sector)**.

In the binational agenda, driven by the CSF, a relationship of greater autonomy regarding national priorities is identified. Although the working plan of the CSF calls for actions aligned to the national health programs of both countries, the health needs specific to the border context and population tend to be prioritized over those of the national agenda.

*“Well, the* [Federal] *Secretary of Health tells me that he wants the CSF in Baja California to work on Zika*, [and I respond] *-well, let me consult it to the members, right?- And it turns out that the Secretary of Health* [of Baja California] *is also a member* [of the CSF], *and he says: –No, I have other priorities, my number one is tuberculosis, not Zika because people are dying of tuberculosis and I want this to be worked bi-nationally because we have drug users who come from* [US] *to the Rehabilitation Centers in Baja California, and then the number of cases of tuberculosis increases.“***(Informant F, Health Sector)**.

#### Foreign Affairs Sector

The recognized national-level actors to consult or negotiate decision-making at the global agenda are the HS, SRE, and SHCP. As a rule, the governmental entities make the decisions as they represent the commitments and interests that affect the whole nation, and these decisions must be taken at the highest level by the corresponding federal authorities. Civil society and the non-for-profit private sector are recognized as having little influence, if any, on decision-making processes at the national level. However, one of the informants points out that the process of arriving at a consensus is very complex because there are many economic, political, and ideological interests.

*“We are trying to increasingly build more transparent and plural processes towards all these sectors* [including] *the entities that have some legitimate interest in this, such as NGOs and the industry, so that they can be heard. We listen to them and we decide what to do with what we are told* (…) *NGOs are influential, with certain groups of countries. Some countries are not so receptive to the voice of civil society. There are political interests, there are ideological visions, and there are economic interests.“ ***(Informant D, Foreign Affairs Sector).**

The representatives of Mexico in international organizations, for example, in Geneva and particularly at WHO, remark that the opinions or recommendations of a technical nature coming from the SRE and the HS are heard. However, the interview data suggest that this cooperation and consultation with the HS seems to be just rhetorical. The incorporation of health issues in international cooperation with South and Central America countries is guided by the 2011 Law of International Cooperation for Development of Mexico. This situation involves a double agenda, one related to US border matters and another linked to Mesoamerica issues.

*“From the perspective of how to incorporate any topic into the cooperation portfolio, including health, the 2011 Law of International Cooperation for Development of Mexico, which is established by the Mexican Agency for International Development Cooperation, defines two regional priority areas for Mexico: Central America, and Latin America as a whole, but with the emphasis on Central America and the Caribbean. So, we work with the sense of being able to build a robust portfolio of cooperation from a first instance of the demands of the countries, and that is where we are identifying opportunity niches, including health, specifically in the Mesoamerica Project.“***(Informant B, Foreign Affairs Sector)**.

#### NGO Sector

Depending on the subject, NGOs establish a dialogue and team up in coalitions with other organizations, such as independent media, that serve as key actors to disseminate information about health issues or initiatives. According to one interviewee, NGOs perceive a lack of knowledge of how health issues should be handled reciprocally by the FAS and HS. For instance, how health issues presented to diplomats should be managed or which diplomatic strategies the HS personnel should be familiar with.

*“At least what I have seen in both Geneva and* [UN in NYC] *is that* [personnel from the FAS] *are not as specialized in health issues as such. A single person or a team has to analyze different issues and rely on the links with the official representations from the* [HS] *to strengthen the participation of Mexico* [in global fora]. *They have much less experience in comparison with trade, economics, or environmental issues* (…) *I believe that health is still not seen as a priority area within the representatives of Mexico in international organizations.“***(Informant I, NGOs)**.

From the NGOs’ perspective, Mexico as a global actor is prone to sign treaties, conventions, and agreements worldwide but does little to broadly disseminate its international commitments domestically for an effective political impact. Consequently, the possibility for civil society to successfully demand concrete actions, accountability, or transparency from the government is limited. Many of the signed agreements that are not binding, especially those with international organisms such as WHO, are often not implemented at the national level.

*“There is a huge distance between the signature of an international agreement and the implementation of public policy at the local level. There is also a need to strengthen accountability mechanisms, including sanctions, so what is agreed at the international level is implemented at the local level* (…) *The participation of civil society remains a mystery, let us say there are meetings with an area of the SRE, but finally, the Presidency, who coordinates the follow-up work on the agenda, is completely closed to the participation of civil society.“***(Informant G, NGOs)**.

From the perspective of NGO informants, the interests of private companies, such as the pharmaceutical industry, can also influence the actions of governmental bodies like the Federal Commission for the Protection Against Sanitary Risk (COFEPRIS) or intersectoral institutions like the Mexican Observatory of Non-Communicable Diseases (OMENT). It should be noted the existence of vested interests in some of these governmental bodies. At the time of our interviews, for example, half of the OMENT members represented the industry.

As part of the political visibility strategies supported by NGOs, the informants highlighted public campaigns, the use of social networks, and the identification of windows of opportunity within the broad context and the processes the country is going through.

*“*[HS] *has taken a position to defend the interests of the big corporations* (…) *When in 2013* [the President] *created the National Strategy for the Prevention and Control of Overweight, Obesity, and Diabetes* (…) *the Secretary of Health was sponsored by Nestlé and created a special forum* [represented] *by the industry* (…) *In fact, there was no representation of the National Health Institutes there, but many industry businesses have a sit on it. HS only participates in those* [issues] *that do not intersect with the interests of large corporations.“***(Informant H, NGOs)**.

### Impact of multilateral negotiations on decision-making at the national level

The implementation of multilateral agreements in Mexico is a complex process that can fail due to political, security, or commercial interests, and so the impact that multilateral negotiations may have at the local level is limited. Some sectors, both private and governmental, might be negatively affected by a decision aimed to protect population health that was taken at a binational level (i.e., food labeling between Mexico and the US), and governance processes may develop as a consequence, including political and civil society pressure or lobbying.

#### Health Sector

According to some informants, the HS’s role in these circumstances is to reduce the pressure that actors outside the sector can have in order to maintain decisions oriented towards improving population health and well-being.

*“Yes, we have sought to ensure that the HS is not influenced by these private actors, which basically are the pharmaceutical industry, but also other non-state actors* (…) *civil society organizations, sometimes they want to impose their* [particular interests like] *HIV/AIDS. We started to receive very strong pressure from the industry. Many times* (…) *these agreements offer the possibility to be gradually implemented, according to national regulations.“***(Informant E, Health Sector)**.

Negotiation processes become more complex when an agreement signed at the international level affects not only local, non-governmental actors, but also other governmental or private sectors. Such is the case of the WHO Framework Convention on Tobacco Control, where the HS advocates against harm to health and the Secretariat of Economy defends producers’ interests.

*“Intra-institutional coordination is always challenging.* [In such cases] *we ask the FAS to take the lead in solving any problem that can happen. Many times we end up opposed. The Secretariat of Economy supported the tobacco producers in Nayarit, and we say that smoking is harmful to health* (…) *The work of coordination is entirely led by the SRE* [and] *it is very complicated to agree* [with the other Secretariats of] *Agriculture, Economy, and Health.“***(Informant E, Health Sector)**.

#### Foreign Affairs Sector

The role of industry and competing sectoral interests as barriers to GHD implementation was also mentioned by respondents from the FAS. One informant provided an example of the difficulties encountered in attempts to follow WHO recommendations at the national level to regulate the ultra-processed food trade in Mexico.

*“The* [HS] *spoke with the deputies and the senators* [mentioning] *a serious problem of morbid obesity in the country and the health system cost is brutal* (…) *the legislators must approve some laws to forbid, for example, junk food selling in the schools, but* [some senators and representatives] *received money from* [junk food industry] (…) *There was a decision made two or three years ago that* [public drinking fountains in primary schools] *must be made available, but the initiative disappeared, I do not think it was accidental* (…) *and we were not complying with the decisions that were approved at international level.“***(Informant A, Foreign Affairs Sector)**.

Ultimately, the implementation of recommendations at the national level depends on the decisions of congress members who are often influenced by corporate lobbying practices. At this level of decision-making, the informant recognized a limited power of influence coming from the FAS.

#### NGO Sector

Informants from the NGO sector mentioned that sometimes the HS is reluctant to support the commitments acquired at the regional level fully. This is more frequent when the implementation of global actions conflict with competing interests at the national level, such as those of the food industry.

*“At least from the experience that I had with the NGO* Contrapeso (Counterweight), *being a member of the Advisory Council in the OMENT* (…) [in HS or COFEPRIS] *we did not see an openness to recognize the flaws in the subject of labeling, or on the issue of the advertising aimed at children* (…) *I understand to a certain extent the position of* [HS] *where they say they have to have openness and dialogue with all sectors* (…) *However, I think that that openness is in excess, in terms of discussing which policy can be modified, which policies cannot, obviously end up to a conflict of interest* (…) *if it is an interest in protecting health, or is an economic interest of companies, of industrial sectors* [this complicates the process and generates] *aggressive confrontations.“***(Informant H, NGOs)**.

## Discussion

In this analysis we focused on the experiences of KI from three different sectors involved in GHD processes in Mexico. Although exiguous, some previous literature provides concrete examples of GHD processes in Mexico in its north and south borders, such as migrant’s health or malaria control initiatives. [26] [8] However, to our knowledge, this is the first time that a study analyzing testimonies from key actors on the field, based on a specific reference framework on the intersection between public health and international relations, has been conducted in Mexico.

Our analysis demonstrates that GHD in Mexico is driven hierarchically by the FAS. This high formalization and hierarchy restrict the participation of other sectors, particularly NGOs and private actors, in decision-making processes and limits other state actors, like the HS. In their role as representatives of Mexican foreign policy and under the framework of the national priorities, the high-ranking officials usually decide on issues, actions, and signing of health-related international agreements. This type of leadership, which tends to be rigid, is congruent with a realist approach to international relations where actions seek to preserve national interests, [2] even if it entails setting boundaries to other domestic actors’ actions, as our findings suggest. Despite having a fundamental leadership in the binational health agenda and introducing migrant’s health as a global health concern for regional integration, the role of the HS in GHD processes seems to be limited to just technical assistance for the FAS, particularly in multilateral agenda-setting or negotiations. This situation, at least for the cases presented in our findings, seems to be consistent with the high-low politics rationale, where health tends to be categorized as an “apolitical” or scientific matter, only becoming relevant to international affairs when connected with commercial or security concerns. [4, 13] Furthermore, the need to strengthen technical capacities in health within the FAS and international relations within the HS could provide an opportunity to improve the GHD initiatives in which Mexico gets involved in the future.

This hierarchical relationship between the FAS and the HS has had exceptions. One example was the joint participation of the Sub-secretary of Health and the Secretary of Foreign Affairs in the 2020 G20 meeting, where the SARS-Cov-2 pandemic was part of the agenda as a pressing matter for global health. However, the official SRE media release of the event did not mention the participation of the Mexican Sub-secretary of Health in the summit. [27] This omission could be indicative of a tendency for keeping hierarchical sectoral relationships and power influence untouched. The FAS, nonetheless, takes credit for its advocacy in securing SARS-Cov-2 vaccines for Mexico, effective November 2020. [28] This example shows how Mexico’s COVID-19 pandemic response is primarily managed by the FAS, leaving behind the HS leadership. This marginal role of the HS during the pandemic can be partially attributed to the cumbersome bureaucratic nature of the HS and its capacity being fully taxed while coping with the pandemic.

Another characteristic of GHD in Mexico is the lack of formal coordination mechanisms between the FAS and the HS to deal with issues that require inter-sectoral collaboration. Both the HS and the FAS recognize that there are no institutionalized processes for the inter-sectoral coordination of health issues that require a diplomatic response or, conversely, of foreign policy situations that entail health consequences. In the absence of these mechanisms, KI from both sectors pointed towards ad-hoc experiences where communication was crucial for action on health at a global scale, such as the CSF. Arguably, the CSF has a certain degree of autonomy for prioritizing global health issues and in the execution of transboundary actions (programs and policies). The CSF could be considered as a specialized bureaucracy that exercises global health diplomacy on a daily basis. As described by participants, most of the CSF actions are best understood through constructivist theories of international relations, in which goal attainment is mainly based on recognition of shared values, this case being development and health promotion. [2]

The power of influence for GHD decision-making of non-governmental actors, such as NGOs, is limited, displaying an asymmetry of power in framing health issues within the foreign policy agenda. As a response, according to KI testimonies of the NGO sector, they actively seek for political windows of opportunity and have adopted lobbying, advocacy, and networking strategies, including the use of evidence from academic research, to oppose the adoption of public policy decisions that can be harmful to health. [14] In addition to advocacy, monitoring, and denouncing, NGOs lobby for improvements in government accountability for decisions taken in the global arena, especially those non-legally binding agreements or recommendations that Mexico may adhere to. All these strategies correspond to a practical expression of GHD that needs to be strengthened so that this sector can play a more influential role in global decision-making. A fruitful example of this engagement is the recent change in the legislation of the Mexican Official Norm on food and non-alcohol beverages. As a result, from October 2020 onwards, all such traded products must include a frontal label warning if they are considered nutritionally unhealthy or harmful to children. [29] This positive outcome resulted primarily from the constant pressure that the NGO sector has made to position the issue in the public health agenda, joined by other sectors like the academia. [30]

One interesting finding from our analysis is the “opposing narratives” between the HS and the NGO regarding their relationship with private actors (such as pharmaceuticals and industry). KI from both sectors mentioned that their actions prevented the imposition of industry interests in decision-making processes. The NGO sector constantly points out that government decisions are often influenced by private interests, such as tobacco or sugar industries. Conversely, HS not only mentions that they usually struggle with the pressure of private actors, but also try to prevent NGOs from imposing their particular agenda. In any case, both narratives suggest acknowledging each other sectors about the power of influence of GHD processes. Another opposing narrative regarding the intersectoral collaboration between FAS and HS can be identified when a KI from the HS mentions “good communication” between both sectors in GHD process and NGO stresses the separation and lack of trust between “institutions and their officials”.

## Conclusions

Our goal in this study was to provide evidence to advance existing knowledge on the links between the health and foreign affairs sectors in Mexico and, to some extent, in the Latin American region. This analysis indicates that the processes of diplomacy for global health in Mexico are carried out in a context of asymmetry of power in which government actors possess the main decision-making capacity vis-à-vis other non-governmental entities. Opportunities are identified for the generation of formal communication mechanisms for GHD at the intersectoral government coordination. One first step towards creating these mechanisms lies in the capacity strengthening and training of the FAS and HS personnel in topics such as global public health and sanitary risks (including those related to trade) and foreign policy and diplomacy, respectively. Our findings and recommendations support similar mechanisms that have been proposed elsewhere as a way to professionalize the field of GHD. [31]

Our study is not exempt from several limitations. Our results derived from interviews of carefully chosen KI involved in GHD processes and the purposive sampling method used, along with a small number of interviewed subjects, do not allow us to make generalizations on the dynamics of GHD processes. Nonetheless, these findings provide a solid starting point for developing a research agenda on GHD processes in Mexico and the Americas region.

A final note worth mentioning is that the onset of COVID-19 pandemic suggests a return to more bilaterally and regionally focused forms of GHD, with a relative lack of global health cooperation to address the root causes and deal with the consequences of the pandemic. [32] This can best be seen in the global failure to develop an equitable distribution system for vaccines. As we noted initially, Mexico’s GHD has been strongly influenced by various bilateral and regional policy initiatives, and as such, Mexico is well-positioned to succeed in a new GHD system that is more bilaterally and regionally focused. As the COVID-19 pandemic changes the patterns of GHD activities, a shift to more regional coordination in GHD seems to be a better way to successfully respond to the many challenges faced during this international emergency. This shift will allow Mexico to use its regional leadership position to make meaningful contributions to GHD, and play a leadership role in the Americas, provided it addresses its own institutional limitations to become an influential actor in the realm of GHD.

## Supplementary information

**Additional file 1**

## Data Availability

The datasets generated and analyzed during the current study are not publicly available to ensure confidentiality and anonymity of research subjects, but are available from the corresponding author on reasonable request.

## Abbreviations

COFEPRIS: Federal Commission for the Protection against Sanitary Risk
CSF: US-Mexico Border Health Commission
FAS: Foreign Affairs Sector
GHD: Global Health Diplomacy
HS: Health Sector/Secretariat of Health
KIs: Key informants
NGOs: Non-Government Organizations
OMENT: Mexican Observatory of Non-Communicable Diseases
SHCP: Secretariat of Finance and Public Credit
SRE: Secretariat of Foreign Affairs
US: United States of America

## Footnotes

1 The Mexico-United States Border Health Commission is a binational organization, created in July 2000 through an agreement between the governments of Mexico and the United States, with the purpose of identifying and assessing health problems that affect the border population, as well as facilitate actions for their attention. (https://www.saludfronterizamx.org/en/)

2 As a result of this controversy, the frontal labeling was modified and implemented in Mexico in 2020.

## References

[1] Kickbusch I, Silberschmidt G, Buss P (2007). Global health diplomacy: the need for new perspectives, strategic approaches and skills in global health. Bulletin of the World Health Organization.

[2] Ruckert A, Labonté R, Lencucha R, Runnels V, Gagnon M. Global health diplomacy: A critical review of the literature. Social science & medicine (1982). 2016;155:61-72. 10.1016/j.socscimed.2016.03.004 26994358

[3] Tobar S, Buss P, Coitiño A, Kleiman A, Fonseca LE, Rigoli F (2017). Health diplomacy: strengthening the international relations offices of health ministries of the AmericasDiplomacia da saúde: fortalecimento dos escritórios de relações internacionais dos ministérios da Saúde das Américas. Rev Panam Salud Publica.

[4] Labonté R, Gagnon ML (2010). Framing health and foreign policy: lessons for global health diplomacy. Globalization and Health.

[5] Collins-Dogrul J. Managing US-Mexico “border health”: an organizational field approach. Social science & medicine (1982). 2006;63(12):3199-211. 10.1016/j.socscimed.2006.07.031 16987573

[6] Comisión de Salud Fronteriza México-Estados Unidos. ¿Quiénes somos? Tijuana: Comisión de Salud Fronteriza México-Estados Unidos; nd [August 14, 2020]. Available from: https://saludfronterizamx.org/quienes-somos/.

[7] Instituto de los Mexicanos en el Exterior. ¿Qué hacemos? Mexico City: Instituto de los Mexicanos en el Exterior; nd [June 3, 2020]. Available from: https://www.gob.mx/ime/que-hacemos.

[8] Rangel Gomez MG, Tonda J, Zapata GR, Flynn M, Gany F, Lara J (2017). Ventanillas de Salud: A Collaborative and Binational Health Access and Preventive Care Program. Front Public Health.

[9] Hernández-Prado B, Kestler E, Díaz J, Walker D, Langer A, Lewis S, et al. Perfil situacional y estrategias de intervención en la región mesoamericana en el área de salud materna, reproductiva y neonatal. salud pública de méxico. 2011;53:s312-s22. 22344376

[10] Friel S, Gleeson D, Thow A-M, Labonte R, Stuckler D, Kay A, et al. A new generation of trade policy: potential risks to diet-related health from the trans pacific partnership agreement. Globalization and Health. 2013;9(1):46. 10.1186/1744-8603-9-46 PMC4016302 24131595

[11] Labonté R, Almeida C, Buss P, Gagnon ML, Lencucha RA, Muñoz F, et al. Global Health Diplomacy: An explanatory multi-case study of the integration of health into foreign policy. Canadian Institutes of Health Research; 2014.

[12] Fidler DP (2010). Negotiating Equitable Access to Influenza Vaccines: Global Health Diplomacy and the Controversies Surrounding Avian Influenza H5N1 and Pandemic Influenza H1N1. PLOS Medicine.

[13] Fidler DP. Assessing the foreign policy and global health initiative: the meaning of the Oslo process: Chatham House London; 2011.

[14] Gagnon ML, Labonté R (2013). Understanding how and why health is integrated into foreign policy - a case study of health is global, a UK Government Strategy 2008–2013. Globalization and Health.

[15] Simmons BA, Elkins Z (2004). The Globalization of Liberalization: Policy Diffusion in the International Political Economy. American Political Science Review.

[16] Krasner SD. Are Bureaucracies Important? (Or Allison Wonderland). Foreign Policy. 1972(7):159–79.

[17] Rasanathan K, Bennett S, Atkins V, Beschel R, Carrasquilla G, Charles J, Dasgupta R, Emerson K, Glandon D, Kanchanachitra C, Kingsley P, Matheson D, Mbabu RS, Mwansambo C, Myers M, Paul J, Radebe T, Smith J, Solar O, Soucat A, Ssennyonjo A, Wismar M, Zaidi S. Governing multisectoral action for health in low- and middle-income countries. PLOS Medicine. 2017;14(4):e1002285. 10.1371/journal.pmed.1002285. 10.1371/journal.pmed.1002285 PMC5404752 28441387

[18] Varvasovszky Z, Brugha R (2000). A stakeholder analysis. Health Policy and Planning.

[19] González G, Pellicer O, Saltalamacchia N. México y el multilateralismo del siglo XXI: reflexiones a los 70 años de la ONU. 1 ed. México: Siglo XXI Editores; 2015 2015. 527 p.

[20] Orozco-Desa MA. Diplomacia parlamentaria. Revista Mexicana de Política Exterior. 2000-2001;62-63:1-19.

[21] Poder Legislativo. Dictamen de las Comisiones Unidas de Relaciones Exteriores Organismos Internacionales, de Relaciones Exteriores; y de Atención a Grupos Vulnerables, por el que se aprueba el Tratado de Marrakech para Facilitar el Acceso a las Obras Publicadas a las Personas Ciegas, con Discapacidad Visual o con Otras Dificultades para Acceder al Texto Impreso. Gaceta del Senado (México); 2013.

[22] Decreto Promulgatorio del Convenio Marco de la OMS para el Control del Tabaco, (25 de febrero de 2005, 2005).

[23] Decreto Promulgatorio del Acuerdo entre el Gobierno de los Estados Unidos Mexicanos y el Gobierno de la República de Guatemala para establecer una Comisión de Salud Fronteriza México-Guatemala, (14 de abril del 2004, 2004).

[24] Ruckert A, Almeida C, Ramírez J, Guerra G, Salgado de Snyder VN, Orozco E, et al. Global Health Diplomacy (GHD) and the integration of health into foreign policy: Towards a conceptual approach. 2021:1-14. 10.1080/17441692.2021.1900318 33736572

[25] Gourevitch P (2009). The second image reversed: the international sources of domestic politics. International Organization.

[26] Chanon KE, Mendez-Galvan JF, Galindo-Jaramillo JM, Olguin-Bernal H, Borja-Aburto VH (2003). Cooperative actions to achieve malaria control without the use of DDT. International journal of hygiene and environmental health.

[27] Secretaría de Relaciones Exteriores. México participa en la Cumbre Virtual de Líderes del G20 sobre la pandemia del COVID-19 Mexico City: Secretaría de Relaciones Exteriores; 2020 [August 14, 2020]. Available from: https://embamex.sre.gob.mx/eua/index.php/es/boletines/1654-mexico-participa-en-la-cumbre-virtual-de-lideres-del-g20-sobre-la-pandemia-del-covid-19.

[28] Forbes. Vacuna contra Covid-19 sería producida en México a partir de noviembre: Ebrard Mexico City: Forbes Mexico; 2020 [August 14, 2020]. Available from: https://www.forbes.com.mx/politica-vacuna-contra-covid-19-seria-producida-en-mexico-a-partir-de-noviembre-ebrard/.

[29] Secretaría de Gobernación. Acuerdo por el cual se establecen los Criterios para la implementación, verificación y vigilancia, así como para la evaluación de la conformidad de la Modificación a la Norma Oficial Mexicana NOM-051-SCFI/SSA1-2010, Especificaciones generales de etiquetado para alimentos y bebidas no alcohólicas preenvasados-Información comercial y sanitaria, publicada el 27 de marzo de 2020. Mexico City: Diario Oficial de la Federación; 2020 [cited 2020 September 7]. Available from: https://www.dof.gob.mx/nota_detalle.php?codigo=5596558&fecha=10/07/2020.

[30] Kaufer-Horwitz M, Tolentino-Mayo L, Jáuregui A, Sánchez-Bazán K, Bourges H, Martínez S (2018). Sistema de etiquetado frontal de alimentos y bebidas para México: una estrategia para la toma de decisiones saludables. Salud Pública de México.

[31] Ariansen AMS, Gloppen S, Rakner L, Johansson KA, Haaland ØA (2020). Time for global health diplomacy. The Lancet.

[32] Fazal TM (2020). Health Diplomacy in Pandemical Times. International Organization.

