# Supplementary material for: Global health diplomacy in Mexico: insights from key actors in the field
Source: Global Health. 2021 Dec 2;17:137. doi: 10.1186/s12992-021-00789-y (PMC8637518; doi:10.1186/s12992-021-00789-y)

**Global Health Diplomacy in Mexico: insights from key actors in the field**

Supplementary material

**Tree code for KIs interviews**


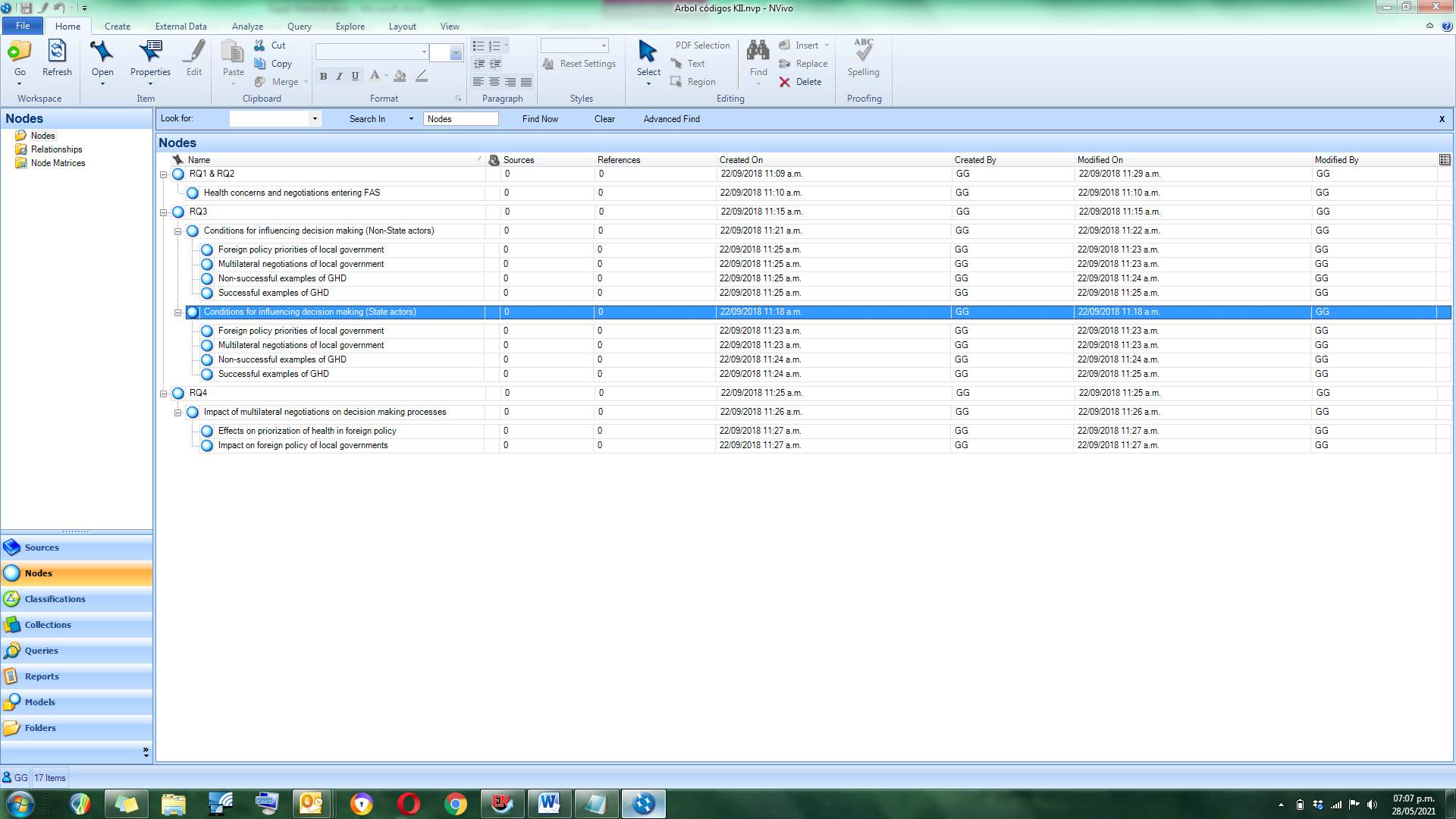

Supplement: Supplementary file 1 — Additional file 1 [file 12992_2021_789_MOESM1_ESM.docx]
